# Increased pulmonary blood flow leads to alveolar dysplasia during the early postnatal developmental stage

**DOI:** 10.1186/s13578-025-01502-x

**Published:** 2025-11-24

**Authors:** He Zhang, Sixie Zheng, Zheng Wang, Yingying Xiao, Yuqing Hu, Debao Li, Qing Cui, Chenxi Liu, Yiting Xue, Junhua Wu, Sijuan Sun, Lincai Ye

**Affiliations:** 1 Department of Pediatric, The Affiliated Women and Children’s Hospital of Ningbo University, Ningbo, Zhejiang China; 2 Department of Thoracic and Cardiovascular Surgery, Shanghai Children’s Medical Center, Shanghai Jiao Tong University School of Medicine, Shanghai, China; 3 Department of Cardiology, Shanghai Children’s Medical Center, Shanghai Jiao Tong University School of Medicine, Shanghai, China; 4 Department of Pediatric Surgery, Children’s Hospital of Fudan University, National Children’s Medical Center, Shanghai, China; 5 Department of Pediatric Intensive Care Unit, Shanghai Children’s Medical Center, Shanghai Jiao Tong University School of Medicine, 1678 Dongfang Road, Shanghai, 200127 China; 6 Shanghai Institute for Pediatric Congenital Heart Disease, Shanghai Children’s Medical Center, Shanghai Jiao Tong University School of Medicine, 1678 Dongfang Road, Shanghai, 200127 China

**Keywords:** Increased pulmonary blood flow, Animal models, Pulmonary dysplasia, Lung, Congenital heart diseases

## Abstract

**Background:**

Increased pulmonary blood flow (IncPBF), one of the most important features of many children with congenital heart diseases, is well-known as a prerequisite for the induction of pulmonary arterial hypertension. However, due to the lack of neonatal mouse models of IncPBF, it remains largely unknown how IncPBF affects postnatal lung development.

**Methods and results:**

A neonatal mouse model of IncPBF was created via abdominal aorta and inferior vena cava fistula microsurgery at postnatal day 7 (P7) and verified by abdominal ultrasound and cardiac ultrasound. Hematoxylin–eosin staining demonstrated that at P14, the number of alveoli was significantly reduced in the IncPBF group compared with the sham group. Immunostaining further confirmed the results, showing that the markers of alveoli type 1 (AT1), alveoli type 2 (AT2), and endothelial cells were significantly reduced in the IncPBF group compared with the sham group. Moreover, RNA-sequencing analysis demonstrated a substantial difference of gene expression profile between IncPBF and sham lungs, and many gene ontology terms or reactome enrichment that are associated with normal alveolar development and pulmonary function, such as angiogenesis, cell migration, and lipid metabolism, were downregulated. Mechanistically, suppression of Mfap5-positive myofibroblasts or Shh-Gli1 signaling could ameliorate IncPBF-induced alveolar hypoplasia.

**Conclusions:**

IncPBF led to alveolar dysplasia during the early developmental stage, and a neonatal mouse model of IncPBF was successfully created. This study introduced a platform for understanding IncPBF-associated pediatric diseases.

**Supplementary Information:**

The online version contains supplementary material available at 10.1186/s13578-025-01502-x.

**Central Message**

- Increased pulmonary blood flow (IncPBF) leads to early alveolar dysplasia.
- A neonatal mouse model of IncPBF was introduced.

**Perspective Statement**

This study introduced a platform for understanding IncPBF-associated pediatric diseases, such as alveolar dysplasia and pulmonary arterial hypertension.

## Introduction

Increased pulmonary blood flow (IncPBF), one of the most important hemodynamic characteristics of many kinds of pediatric congenital heart diseases (CHDs), such as ventricular septal defect (VSD), patent ductus arteriosus (PDA), and atrial septal defect (ASD), has long been recognized as a prerequisite for the induction of pulmonary arterial hypertension (PAH) in pediatric patients with CHDs, who have impaired respiratory function and high mortality [1–5]. Using adult animal models, IncPBF-induced remodeling of pulmonary small blood vessels has been well documented [1–4]. However, to the best of our knowledge, it has not yet been explored how IncPBF contributes to alveolar development because of the lack of neonatal IncPBF mouse model.

More than 90% of alveoli in humans form between 0 and 7 years, while most alveoli in rodents form between postnatal days (P) 0 and P18 [1, 6, 7]. Temporal dynamics of the developing lung transcriptome in three common inbred strains of mice reveals that postnatal alveolar development can be divided into four stages, namely alveolar development stage 1 (ALV1, P0–P3), ALV2 (P4–P7), ALV3 (P9–P12), and ALV4 (P13–P18) [8, 9]. The development of alveoli in adult animals has already been completed, making adult animal models inappropriate for the investigations of alveolar development. About 70% of pediatric PAH cases are caused by IncPBF [10, 11]. Adult efficient PAH-relieving drugs fail to improve the outcomes of pediatric patients with PAH. Currently, there is no cure for most cases of pediatric PAH [12, 13]. Therefore, it is possible that the underlying mechanisms that account for IncPBF-induced remodeling of pulmonary small blood vessels in pediatric patients are different from those in adult patients. Indeed, a recent study that combined genomic, epigenomic, and biophysical methods has revealed that transient myofibroblasts play a crucial role in the formation of alveoli [14]. It is possible that IncPBF-induced remodeling of pulmonary small blood vessels and IncPBF-induced formation of alveoli are interconnected by transient myofibroblasts. A neonatal mouse model of IncPBF would undoubtedly help resolve these concerns.

Gene-modified CHD animals typically die before birth. The only possible way to construct a neonatal mouse model of IncPBF is via microsurgery. Performing microsurgery on neonatal mice is challenging because of the limited operation time and surgical space [1, 6, 15, 16]. We have been working on microsurgery for nearly 10 years and have previously performed abdominal aorta and inferior vena cava fistula (ACF) microsurgery on P7 neonatal mice to induce a left-to-right shunt, which produced neonatal right ventricular volume overload [17–20]. In theory, a left-to-right shunt, just like in PDA and ASD, should produce IncPBF. In addition, ACF surgery has been performed on adult rats to induce PAH [21]. However, more solid data are needed to verify whether ACF microsurgery induces neonatal IncPBF and whether IncPBF affects postnatal lung development. In this study, we used various molecular biology techniques to demonstrate that ACF induces IncPBF and that IncPBF leads to alveolar dysplasia.

## Materials and methods

### Animals

P7 C57/BL6 pups (male or female), bred at the Animal Center of Shanghai Children’s Medical Center, were used in this study. The room was maintained with a 12-h/12-h day/night cycle at a temperature of 21–23 °C.

### Neonatal ACF surgery

The surgical protocol for fistula creation was modified from a previously described method [17–20]. Briefly, at P7, neonatal mice were anesthetized with 4% isoflurane. The pups were placed in the supine position and a midline laparotomy was performed to expose the abdominal aorta (AA) and the inferior vena cava (IVC) (Supplemental Fig. S1A-B). Under microscopic guidance, an 11–0 nylon suture needle (diameter = 0.07 mm) was used to create a fistula by puncturing from the AA into the proximal segment of the IVC at an angle of 30° to 60° (Supplemental Fig. S1B). Successful fistula formation was confirmed by immediate swelling and visible mixing of arterial and venous blood within the IVC. Hemostasis was achieved by applying compression with cotton swabs for 20 s. The abdominal wall was then sutured closed, and the mice were placed on a heating pad maintained at 37 °C until their skin color returned to pink. After full recovery from anesthesia, all of the pups were returned as a group to their mother. The entire procedure was completed within approximately 15 min. Sham-operated mice underwent identical procedures except for the vascular puncture step.

### Ultrasound

At P13, the prepubertal mice were anesthetized with 1.5%–2.0% isoflurane for ultrasound examination. Fistula and pulmonary artery (PA) flow were analyzed with a Vevo 2100 imaging system (Visual Sonics, Toronto, ON, Canada). Using pulsed-wave Doppler mode, we measured blood flow signals in the PA, including the velocity time integral (PA-VTI), diameter of the PA valve (D), pulmonary arterial acceleration time (PAT), and right ventricle (RV) ejection time (RVET). We calculated the RV stroke volume (RVSV, mL) and RV systolic pressure (RVSP, mm Hg) using the following formulas [20, 22]: RVSV [mL] = 1/4 × πD^2^ × PA-VTI; RVSP [mm Hg] =  − 83.7 × PAT/RVET − index + 63.7.

### Tissue preparation

At P14, the lungs of the prepubertal mice were collected for hematoxylin and eosin (H&E) staining, immunofluorescence, and RNA-sequencing analyses. The lungs were perfused with phosphate-buffered saline (PBS) first to clear blood from the vascular bed, followed by fixation with 4% paraformaldehyde. Subsequently, the tissues underwent dehydration, embedding in paraffin, and sectioning.

### H&E staining

Alveolarization was evaluated via H&E staining using the H&E staining kit (Solarbio, Shanghai, China) in accordance with the manufacturer’s instruction. Radial alveolar counts (RACs) and mean linear intercepts (MLIs) were used to quantify alveolarization in line with previous publications [15, 16]. Briefly, RACs were determined by the number of alveoli that transversed a perpendicular line that was drawn from the center of a terminal bronchiole to the edge of the alveolar septum or pleura. To assess MLIs, grids consisting of horizontal and vertical lines were positioned on the image, and the total number of times the borderline intersected with the alveoli and the total length of the grids were recorded. Specifically, MLI was defined as the total grid length divided by the total number of intersections (in μm).

### Immunofluorescence

The paraffin-embedded lung sections were dewaxed in xylene, hydrated using an alcohol gradient, and subjected to antigen retrieval. After incubation in PBS supplemented with 7.5% goat serum and 0.5% Triton X-100 for 1 h at room temperature, the sections were subsequently incubated with primary antibodies (anti-CD31, anti-RAGE, anti-SFTPC, and anti-SMA) (Supplemental Table S1) at 4 °C overnight. The following day, the sections were washed three times with PBST (PBS containing 1% Tween-20), followed by 1-h incubation at room temperature with fluorescent secondary antibodies and DAPI. Finally, the sections were mounted with an antifade medium and sealed with nail polish.

### RNA sequencing

Total RNA was isolated from P14 lungs via PureLink RNA Micro Kit (catalog number 12183016; Life Technologies, Carlsbad, CA, USA) in accordance with the manufacturer's guidelines. Sequencing cDNA library was constructed utilizing NEBNext® UltraTM RNA Library Prep Kit (E7760, NEB, USA). Both library quality and RNA integrity were assessed using an Agilent 2100 Bioanalyzer. Sequencing of the library was performed on the Illumina NovaSeq platform. Raw data were processed through in-house PERL scripts to produce high-quality clean data, which were subjected to differential expression analysis, Gene Ontology (GO), and Kyoto Encyclopedia of Genes and Genomes (KEGG) enrichment analyses. These analyses were performed on the OE cloud platform (https://cloud.oebiotech.com).

### Suppression of Mfap5-positive myofibroblasts or Shh-Gli1 signaling

To achieve lung-specific suppression of Mfap5 -positive myofibroblasts, pscAAV-U6-shRNA (Mfap5)-CMV-EGFP-tWPA (OBIO, China) was administered to mice via intratracheal instillation. Control mice received pscAAV-U6-shRNA (NC2)-CMV-EGFP-tWPA.

Following ACF surgery, mice were treated daily with subcutaneous injections of either cyclopamine (50 mg/kg; MedChemExpress, USA) or vehicle from postnatal day 7 (P7) to P14. The cyclopamine dosage was selected based on a previous report [23].

### Quantitative real-time PCR

Total RNA was extracted from lung tissue using the PureLink RNA Micro Scale Kit (catalog number 12183016; Life Technologies). Quantitative real-time PCR was performed using BeyoFast™ SYBR Green One-Step qRT-PCR Kit (catalog number: D7268M, Beyotime, Shanghai, China) according to the manufacturer’s instructions. *Gli1* primers (Supplemental Table S1) were purchased from Generay Biotech Co., Ltd. (Shanghai, China).

### Western blot analysis

Proteins were extracted from lung tissues using RIPA buffer (P0013B, Beyotime Biotechnology). After quantification by BCA assay, equal amounts of protein were separated by SDS-PAGE and transferred to PVDF membranes. The membranes were blocked with 5% non-fat milk, incubated with MFAP5 primary antibody (Supplemental Table S1) overnight at 4 °C, followed by HRP-conjugated secondary antibodies. Protein bands were visualized using an ECL detection system.

### Statistical analysis

Quantitative data were expressed as the mean ± standard deviation. Differences were tested using Student’s *t* test if the data were normally distributed; otherwise, the rank-sum test was used. P values less than 0.05 were considered statistically significant. The SAS software (SAS 11.2, SAS Institute Inc.) was used for all statistical analyses.

## Results

### Neonatal ACF microsurgery generates a shunt between AA and IVC

**Fig. 1:**
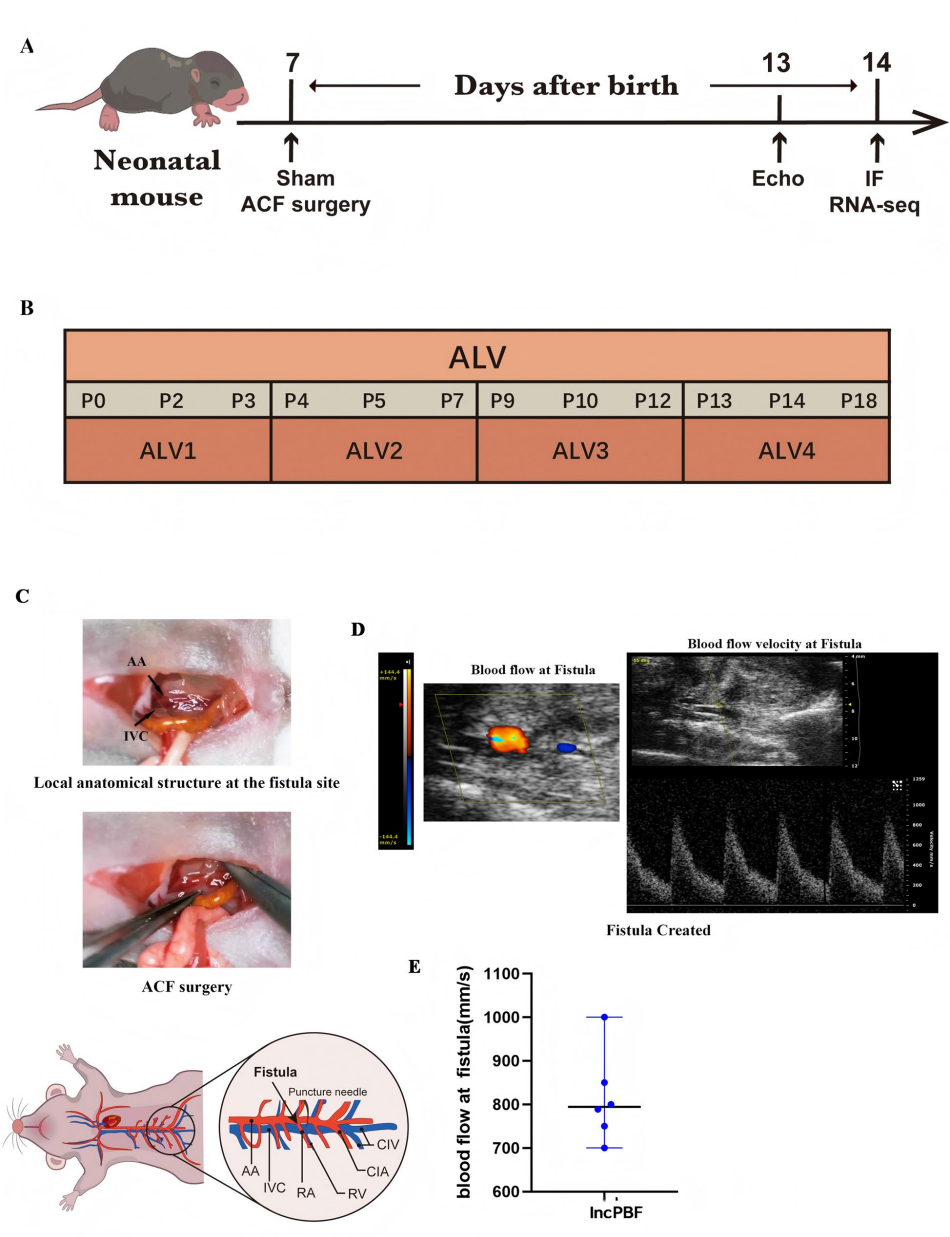
Neonatal abdominal aorta and inferior vena cava fistula (ACF) microsurgery successfully induces a left-to-right shunt. **A** Experimental flowchart. **B** Stages of postnatal alveolar development revealed by transcriptome analysis. **C** Illustration of neonatal ACF microsurgery. **D** Abdominal aortic and venous fistula and blood flow at the fistula. **E** Quantification of blood flow velocity at the fistula

As shown in Fig. 1A–C, we first performed ACF microsurgery on P7 (Supplemental Fig. S1A-B), at ALV2; then, we performed ultrasound at P13 and bulk RNA-sequencing and associated examinations at P14, at ALV4, to investigate whether IncPBF affects alveolar development. The IVC, which served as a negative control, had no pulsatile blood flow (Supplemental Fig. S2A). The AA, which served as a positive control, demonstrated pulsatile blood flow (Supplemental Fig. S2B). There was broad and low pulsatile blood flow at the fistula (Fig. 1D, E) (Video 1). These results suggested that a neonatal shunt between AA and IVC was successfully created, consistent with our previous reports [17–20].

### Neonatal shunt induces IncPBF

**Fig. 2:**
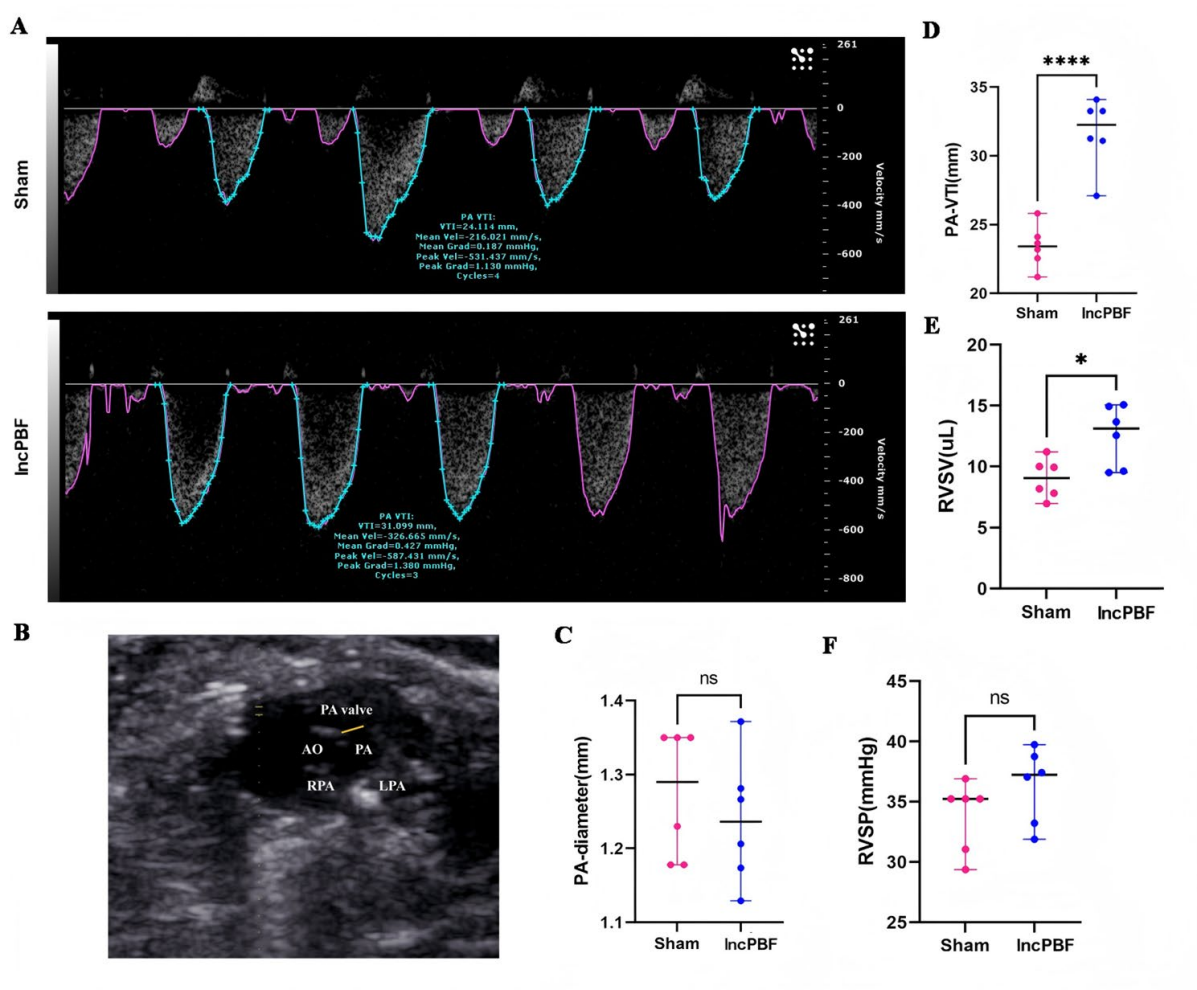
Neonatal shunt generates IncPBF. **A** Representative blood flow imaging at the pulmonary valve. **B** Representative echocardiography at the pulmonary valve. Blue line indicates the PA diameter. **C** Quantification of the PA diameter. **D** Quantification of PA-VTI. **E** Quantification of RVSV. **F** Quantification of RVSP

To confirm that the neonatal shunt produces IncPBF, we performed echocardiography at P13. The results showed that PA-VTI significantly increased in the ACF group (Fig. 2A, B), and there were no differences in the PA diameter (Fig. 2C, D) between the ACF group and the sham group. Therefore, RVSV was significantly increased (Fig. 2E), indicating that neonatal shunt produced IncPBF. There were no differences in PAT and RVET between the ACF and sham groups  . Thus, there were no differences in RVSP between the ACF and sham groups (Fig. 2F), suggesting that the effect of IncPBF in 1 week had not yet produced PAH.

### IncPBF leads to alveolar dysplasia

**Fig. 3:**
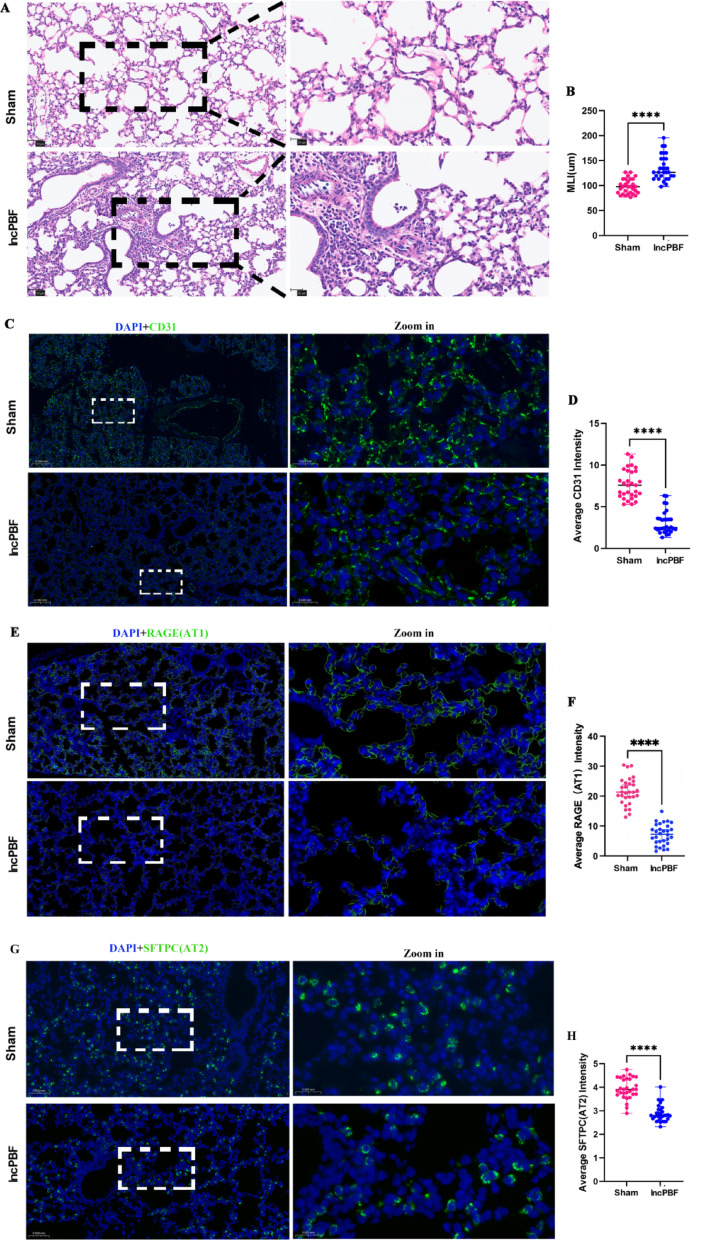
IncPBF leads to alveolar dysplasia. **A** Representative H&E staining of alveoli in the sham and IncPBF groups. **B** Quantification of MLI. **C** Representative immunofluorescence staining of endothelial cells (CD31). DAPI (blue); CD31 (green). **D** Quantification of average CD31 intensity. **E** Representative immunofluorescence staining of AT1 cells (RAGE). DAPI (blue); RAGE (green). **F** Quantification of average sftpc intensity. **G** Representative immunofluorescence staining of AT2 cells (sftpc). DAPI (blue); sftpc (green). (H) Quantification of average sftpc intensity

We then performed H&E staining to investigate whether IncPBF contributes to alveolar development. The results showed that MLI, indicative of alveolar simplification, was significantly elevated in the IncPBF group compared with the sham group (Fig. 3A, B). To confirm these results, we performed immunofluorescence to examine whether the key cells of alveoli were affected by IncPBF [15]. The results showed that CD31, a marker of endothelial cells, was significantly reduced in the IncPBF group compared with the sham group (Fig. 3C, D). Similarly, Sftpc, a marker of type I alveolar cells (AT1), and RAGE, a marker of type II alveolar cells (AT2), were significantly reduced (Fig. 3E, H). These findings indicated that IncPBF led to prepubertal alveolar dysplasia.

In addition, IncPBF induced persistent alveolar dysplasia at P30 and P60 (Supplemental Fig. S3) and was associated with signs of PAH, including elevated right ventricular stroke volume (RVSV) up to two months after ACF surgery, increased right ventricular systolic pressure (RVSP), and pulmonary valve regurgitation at both 2 and 3 months post-surgery (Supplemental Fig. S4).

### IncPBF alters the gene expression profile of normal alveolar development

**Fig. 4:**
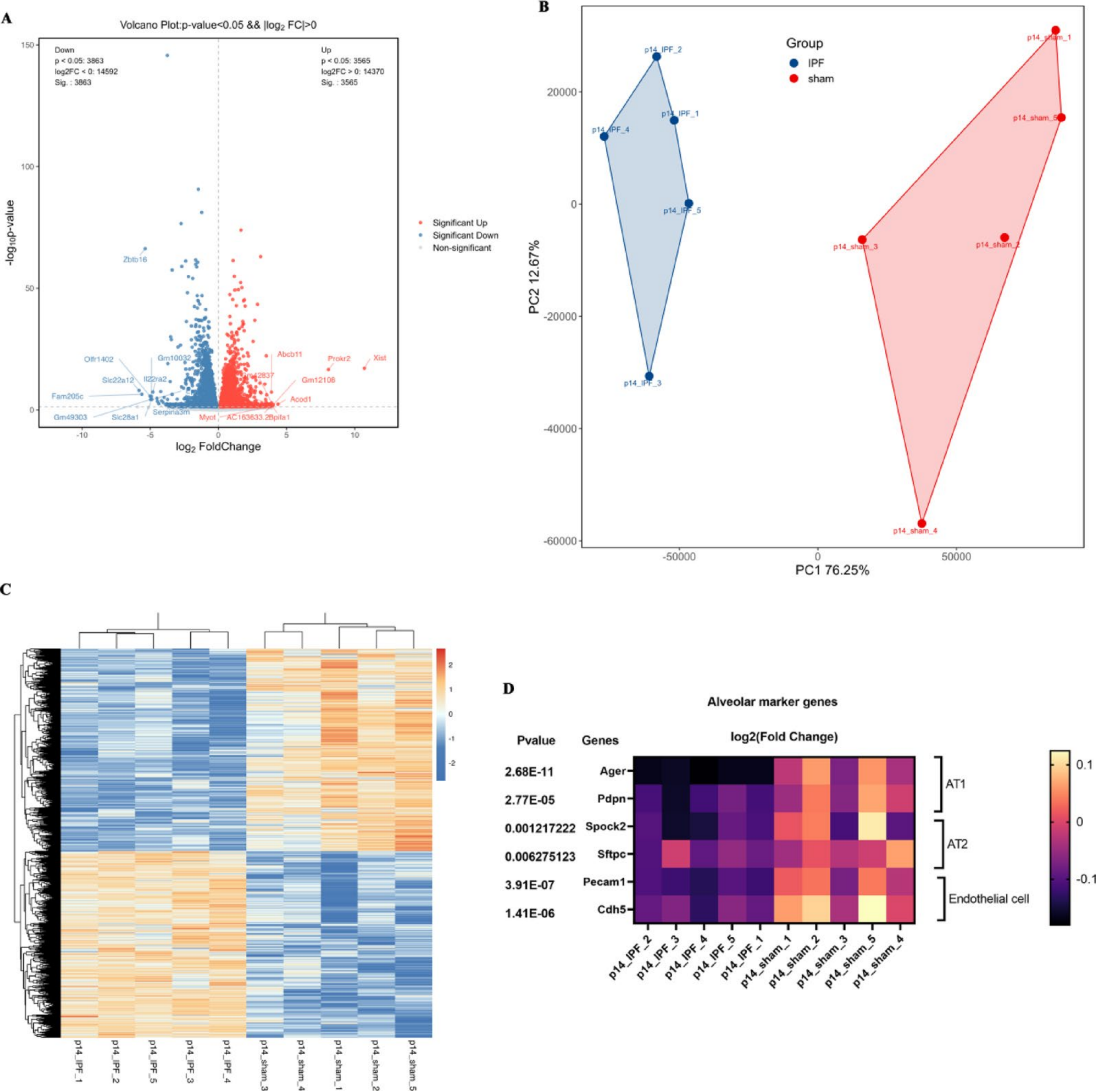
IncPBF leads to transcriptomic changes for postnatal lung development. **A** Volcano plot of differentially expressed genes (DEGs) between the IncPBF and sham lungs. **B** PCA of the DEGs between the IncPBF and sham lungs indicates that the IncPBF lungs are different from the normal lungs. **C** Heat map of cluster analysis of the DEGs between the IncPBF and sham lungs indicates that ACF microsurgery induces highly reproducible lung changes. **D** Heat map shows that the expression of the alveolar marker genes of AT1/AT2/endothelial cells is downregulated due to IncPBF

To further confirm and understand how IncPBF leads to prepubertal alveolar dysplasia, we performed bulk RNA-sequencing of the lungs at P14. The volcanic plot showed that there were 7528 differentially expressed genes (DEGs) between the IncPBF and sham groups, of which 3565 were upregulated and 3863 were downregulated (Fig. 4A). Principal component analysis (PCA) of the DEGs showed that the individual lungs in the IncPBF group were similar to each other but differed noticeably from the lungs in the sham group (Fig. 4B). Likewise, the heat map of cluster analysis demonstrated that the individual lungs in the same group were similar but differed noticeably from the lungs in the other group (Fig. 4C). Detailed analysis of the heat map of cluster analysis revealed that the marker genes of AT1/AT2/endothelial cells were significantly downregulated (Fig. 4D). These results demonstrated that IncPBF microsurgery was highly reproducible and that IncPBF altered the gene expression profile of normal alveolar development.

### IncPBF downregulates the key biological processes and pathways required for normal alveolar development

**Fig. 5:**
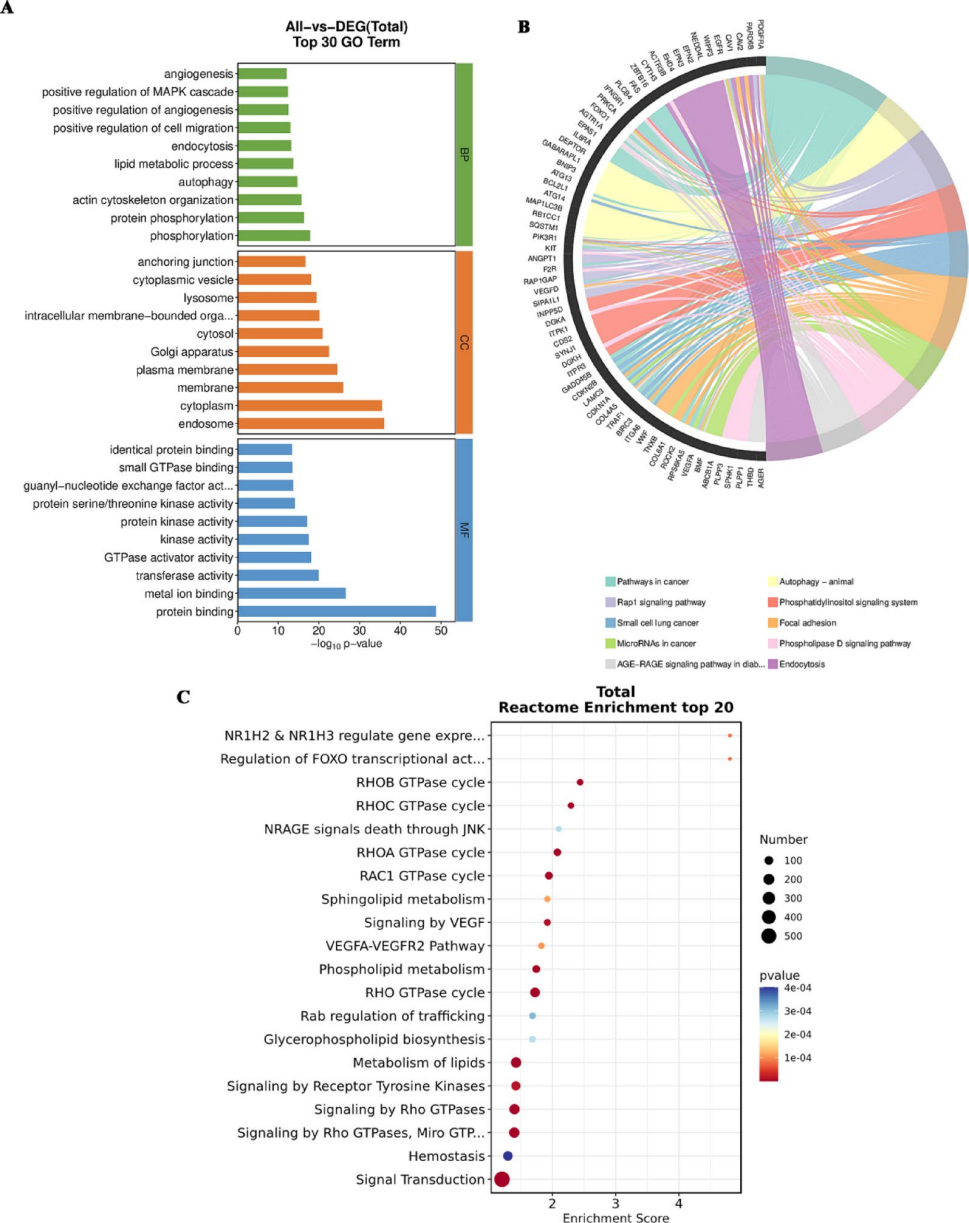
Enrichment analysis of the downregulated DEGs between the IncPBF and sham lungs reveals that the key pathways of postnatal lung development are impaired due to IncPBF. **A** Top 30 enriched GO terms. **B** Top 10 enriched KEGG pathways. **C** Top 20 enriched reactomes

To understand how normal alveolar development was damaged by IncPBF, we subjected the downregulated DEGs to GO and KEGG enrichment analyses. The results showed that the top 10 enriched GO terms in biological process (BP) branch were phosphorylation, protein phosphorylation, actin cytoskeleton organization, autophagy, lipid metabolic process, endocytosis, positive regulation of cell migration, positive regulation of angiogenesis, and positive regulation of MAPK cascade (Fig. 5A); the top 10 enriched GO terms in cellular component (CC) branch were endosome, cytoplasm, membrane, plasma membrane, Golgi apparatus, cytosol, intracellular membrane-bounded organelle, lysosome, cytoplasmic vesicle, and anchoring junction (Fig. 5A); and the top 10 enriched GO terms in molecular function (MF) branch were protein binding, metal ion banding, transferase activity, GTPase activator activity, kinase activity, protein kinase activity, protein serine/threonine kinase activity, guanyl-nucleotide exchange factor activity, small GTPase binding, and identical protein binding (Fig. 5A).

Angiogenesis, metabolism, and migration are critical events of late-stage alveolar development. Lipid metabolism is integral to alveolar surfactant synthesis, and alveolar surfactant is released and recovered through vesicles [8, 24–26]. The GO enrichment results suggested that IncPBF may affect alveolar development by influencing angiogenesis, metabolism, and migration. Indeed, phosphatidylinositol signaling system, focal adhesion, and endocytosis were among the top 10 enriched pathways (Fig. 5B); and metabolism of lipids, glycerophospholipid biosynthesis, sphingolipid metabolism, phospholipid metabolism, signaling by VEGF, and VEGFA–VEGFR2 pathway were among the top 20 enriched reactomes (Fig. 5C).

### The role of transient myofibroblasts in IncPBF-induced alveolar dysplasia

**Fig. 6:**
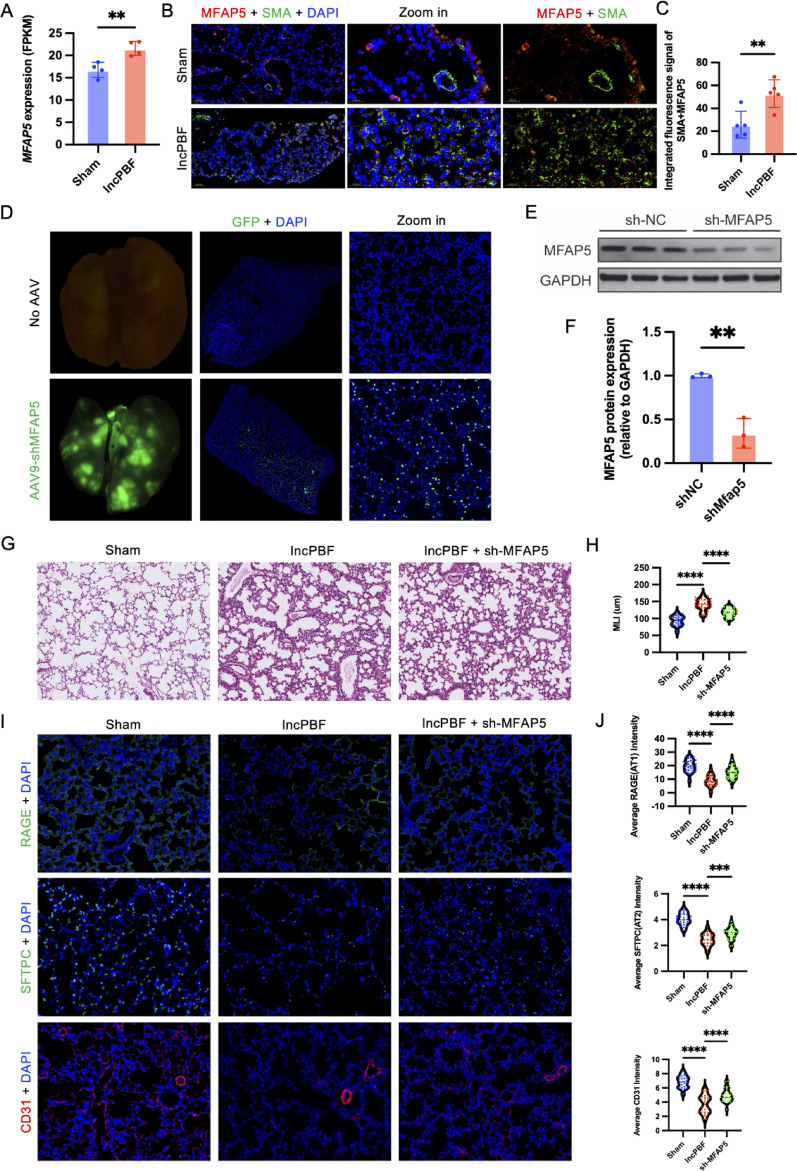
Knockout of Mfap5 ameliorates IncPBF-induced alveolar hypoplasia. **A** IncPBF upregulates Mfap5 expression. **B** Representative immunofluorescence images of Mfap5 and α-SMA double-positive myofibroblasts. Nuclei were stained with DAPI (blue), Mfap5 is shown in red, and α-SMA is shown in green. **C** Quantitative analysis of Mfap5 + /α-SMA + myofibroblasts. **D** Representative images demonstrating successful AAV9-sh-MFAP5 delivery and infection in lung tissues. **E** Representative Western blot bands showing Mfap5 knockdown efficiency. **F** Quantification of Mfap5 protein levels from Western blot analysis. **G** Representative H&E staining of alveoli. **H** Quantification of MLI. (I) Representative immunofluorescence staining for alveolar type I cells (AT1, labeled with RAGE), alveolar type II cells (AT2, labeled with Sftpc), and endothelial cells (labeled with CD31). Nuclei were counterstained with DAPI (blue). RAGE, Sftpc, and CD31 signals are shown in green. (J) Quantification of the mean fluorescence intensity of RAGE, Sftpc, and CD31

IncPBF induced an increased abundance of myofibroblasts (Supplemental Fig. S5). RNA-seq analysis of P14 IncPBF lungs revealed significant upregulation of Mfap5 (p = 0.0361 vs. sham), a known myofibroblast marker (Supplemental Fig. S6 and Fig. 6A), suggesting its potential role as a candidate mediator. To investigate the function of Mfap5 + myofibroblasts in IncPBF-induced alveolar dysplasia, we performed immunofluorescence (IF) co-staining, which confirmed Mfap5 protein overexpression specifically in α-SMA + myofibroblasts in IncPBF lungs (Fig. 6B, C). We then knocked down Mfap5 in IncPBF lungs (Fig. 6D–F) and observed that Mfap5 knockdown rescued alveolarization (Fig. 6G, H) and restored the densities of AT1 cells, AT2 cells, and microvasculature (Fig. 6I–J). These findings support a pathogenic cascade: IncPBF → Mfap5 overexpression in myofibroblasts → impaired alveolarization.

### The role of Shh/Gli1 pathway in IncPBF-induced alveolar dysplasia

**Fig. 7:**
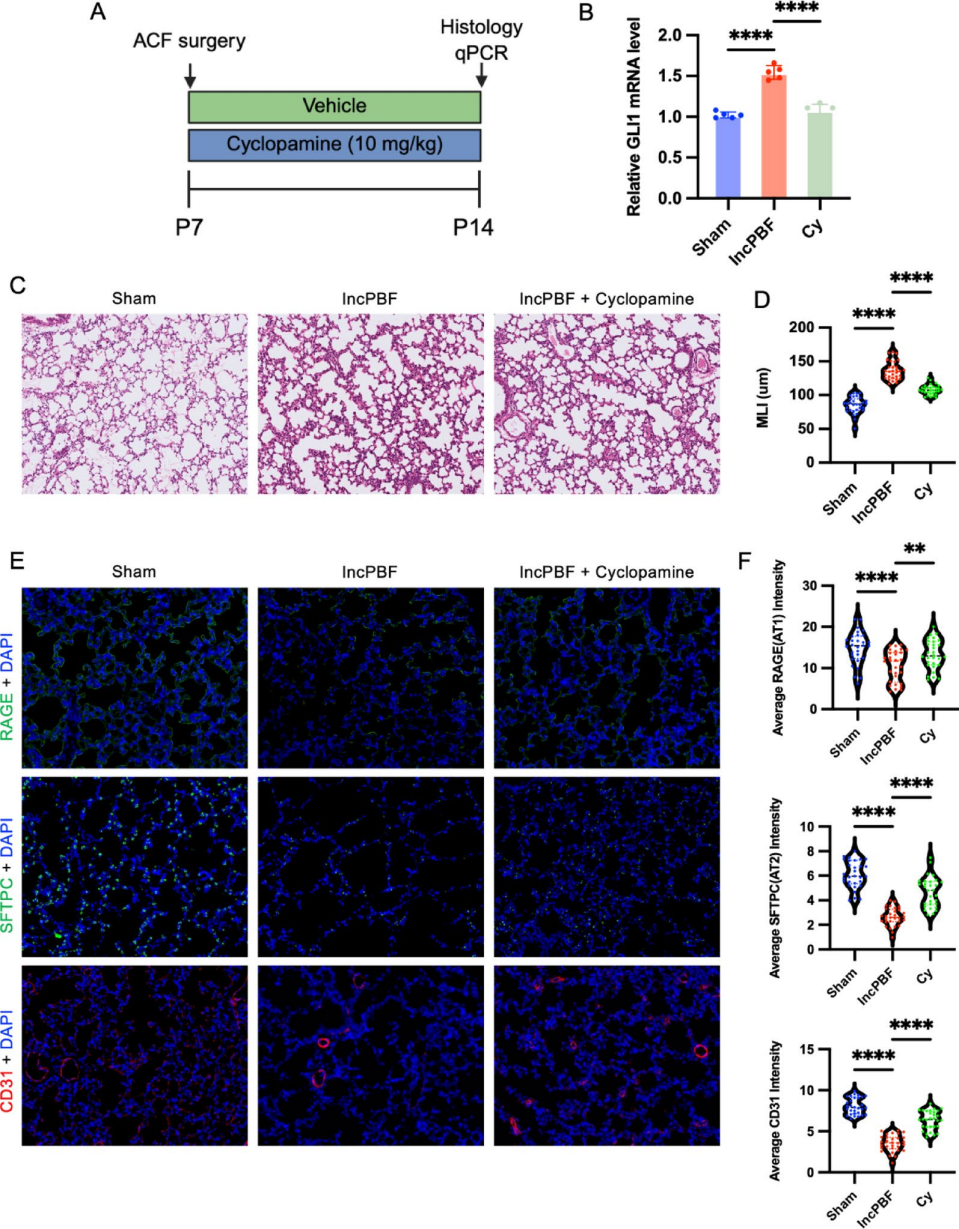
Inhibition of Gli1 ameliorates IncPBF-induced alveolar hypoplasia. **A** Experimental flowchart. **B** Cyclopamine (Cy) inhibits the expression of Gli1. **C** Representative H&E staining of alveoli. **D** Quantification of MLI. (E) Representative immunofluorescence staining for alveolar type I cells (AT1, labeled with RAGE), alveolar type II cells (AT2, labeled with Sftpc), and endothelial cells (labeled with CD31). Nuclei were counterstained with DAPI (blue). RAGE, Sftpc, and CD31 signals are shown in green. **F** Quantification of the mean fluorescence intensity of RAGE, Sftpc, and CD31

AT1 cells secrete Shh, which acts on myofibroblasts and regulates alveolar formation through the transcription factor Gli1 [14]. RNA-seq data revealed that IncPBF upregulated the expression of both Shh and Gli1 (Supplemental Fig. S6). To further investigate the role of the Shh/Gli1 pathway in IncPBF-induced alveolar dysplasia, we treated neonatal IncPBF mice with cyclopamine (Cy) (Fig. 7A, 7), a well-characterized inhibitor of Shh signaling [23]. Cyclopamine treatment downregulated Gli1 mRNA expression (Fig. 7B) and significantly attenuated the increase in MLI in IncPBF mice (Fig. 7C, D), accompanied by partial recovery of CD31, Sftpc, and RAGE expression (Fig. 7E, F). These findings indicate that Shh/Gli1 signaling plays a functional role in IncPBF-induced alveolar dysplasia.

## Discussion

### Recontextualizing IncPBF-induced lung dysplasia

The association between IncPBF and pulmonary vascular remodeling in pediatric CHDs is well-documented [1–5]. While previous adult models have established the role of IncPBF in vascular pathology [1–5], our neonatal mouse model reveals a previously unrecognized developmental dimension: alveolar dysplasia at P14 (Fig. 2) and myofibroblast-driven maldevelopment (Supplemental Fig. S6 and Fig. 6). This finding extends beyond the vascular-centric paradigm proposed by Boucherat et al. [27] and provides the first mechanistic link between hemodynamic stress and alveolar impairment. Importantly, we demonstrate that IncPBF specifically disrupts mesenchymal niches—as evidenced by the functional involvement of Shh/Gli1 signaling (Supplemental Fig. S6 and Fig. 7) and Mfap5 + myofibroblasts (Fig. 6).

### Bridging the translational "valley of death"

No pharmacological cures are currently available for any form of PAH [1–5, 27], with the fact that many preclinically promising drugs have low success during clinical testing—a translational gap referred as the “Valley of Death” (VOD) [1–5, 27]. The alarming near 100% failure rate of PAH drugs in clinical trials [27] stems from critical limitations in conventional disease modeling. Widely used preclinical models (e.g., monocrotaline or sugen /hypoxia induction) exclusively replicate isolated vascular remodeling – a paradigm misaligned with the IncPBF characteristics observed in pediatric CHD patients. In stark contrast, our neonatal IncPBF model achieves three critical advances: First, it uniquely recapitulates the physiological coupling between hemodynamic stress and alveolar development. Where traditional models induce artificial vascular injury in mature lungs, our system mirrors the developmental timeline of CHD neonates by triggering increased pulmonary flow during the alveolarization window (P7–P14). This captures the concurrent emergence of pulmonary arteriopathy (Supplemental Fig. S7) and alveolar simplification (Fig. 3). Second, we identify Mfap5 + myofibroblasts as the unifying node linking vascular and alveolar pathologies. These transient progenitors [14] expand under IncPBF stress (Supplemental Fig. S6), driving a pathogenic cascade through Mfap5 overexpression (Supplemental Fig. S6 and Fig. 6) and dysregulated Shh-Gli1 signaling (Supplemental Fig. S6 and Fig. 7). This positions myofibroblasts as the "upstream driver" (analogous to hepatitis B virus in hepatic inflammation [28]) that initiates vascular remodeling. Third, the model offers a mechanism-informed solution to the translational gap. By targeting the myofibroblast hub (e.g., via Mfap5 knockdown in Fig. 6), we achieved alveolar rescue without hemodynamic interference and concurrent attenuation of vascular remodeling. This demonstrates that decoupling structural pathologies from flow abnormalities is clinically feasible—providing a strategic blueprint to overcome the "Valley of Death" in PAH drug development..

### Clinical implementation framework

This research reveals that IncPBF independently disrupts alveolar formation (reduced alveoli count, downregulated AT1/AT2/endothelial markers) and key developmental pathways (angiogenesis, cell migration, lipid metabolism) before PAH develops. Clinically, this suggests children with high-flow CHDs may suffer from intrinsic pulmonary hypoplasia/dysplasia contributing to respiratory morbidity (e.g., increased susceptibility to infections, prolonged ventilator dependence, bronchopulmonary dysplasia-like phenotypes) alongside PAH. This model enables researchers to identify specific molecular targets (e.g., dysregulated Shh/Gli1 signaling, impaired lipid metabolism for surfactant production) and critical developmental windows (like ALV2-ALV4 in mice, corresponding to early childhood in humans) for potential early interventions aimed at protecting lung development in high-risk infants, potentially improving long-term respiratory outcomes beyond just managing PAH.

This study suggests that transient myofibroblasts, crucial for alveologenesis, might be central to the initiation of IncPBF-induced vascular pathology in children, potentially differing fundamentally from adult PAH mechanisms. By modeling the root hemodynamic cause (IncPBF) in the relevant developmental stage, this platform allows (1) investigation of whether aberrant myofibroblast behavior links alveolar dysplasia and early vascular changes, revealing novel therapeutic targets specific to pediatric IncPBF-PAH, (2) testing of drugs designed not only to reverse vascular remodeling but also to protect or promote alveolar development (e.g., modulating myofibroblast differentiation/function, enhancing specific metabolic pathways like glycerophospholipid biosynthesis), and (3) providing a more physiologically relevant system for preclinical screening of therapies specifically for children with CHD-associated PAH, ultimately aiming to develop effective, disease-modifying treatments that address the unique pathophysiology of pediatric IncPBF.

To better highlight the translational significance of our neonatal IncPBF model, we added a new table (Supplemental Table S2) summarizing how this model overcomes limitations of conventional PAH models and offers unique advantages for studying pediatric pulmonary vascular and alveolar diseases.

### Limitations and directions

However, a lot of work needs to be done to determine whether the transient myofibroblasts are the root cause of pulmonary small blood vessels remodeling. If they are, a question arises as to what their characteristics are and whether they can be the treatment targets for IncPBF-induced PAH. Another question arises as to whether myofibroblasts could be the root cause of other types of PAH. Nevertheless, with the introduction of the neonatal mouse model of IncPBF, several scientific and clinical questions may be answered in the future, e.g., how does IncPBF lead to alveolar dysplasia? what are the underlying mechanisms? and what are the differences between IncPBF-induced pediatric and adult PAHs? The current study introduces a novel platform for exploring IncPBF-associated pediatric diseases.

## Supplementary Information

Below is the link to the electronic supplementary material.

Video 1 A left to right shunt between the aorta and inferior vena cava

Supplementary Material 2

Supplementary Material 3

Supplementary Material 4

Supplementary Material 5

## Data Availability

The data generated in this study are available from the corresponding author upon reasonable request. The bulk RNA-sequencing data have been deposited in the GEO database (https://www.ncbi.nlm.nih.gov/geo) under Accession No. GSE280388.
