# Supplementary material for: Increased pulmonary blood flow leads to alveolar dysplasia during the early postnatal developmental stage
Source: Cell Biosci. 2025 Nov 24;15:161. doi: 10.1186/s13578-025-01502-x (PMC12642049; doi:10.1186/s13578-025-01502-x)
Supplement: Supplementary file 2 — Supplementary Material 2 [file 13578_2025_1502_MOESM2_ESM.docx]

Supplemental Table S1 Primers AND Antibodies

Primers

| Gene |  | Sequence (5'->3') |
| --- | --- | --- |
| Gli1 | Forward | CCAAGCCAACTTTATGTCAGGG |
|  | Reverse | AGCCCGCTTCTTTGTTAATTTGA |

Antibodies

| Name | Company | Catalog No |
| --- | --- | --- |
| CD31 Rabbit Polyclonal Antibody | Beyotime Biotechnology | AF0099 |
| RAGE Rabbit monoclonal Antibody | Abcam, Shanghai | Ab216329 |
| SFTPC Rabbit monoclonal Antibody | Abcam, Shanghai | Ab211326 |
| SMA Mouse monoclonal Antibody | Abcam, Shanghai | Ab7817 |
| MFAP5 Rabbit monoclonal Antibody | Merck, Shanghai | HPA010553 |
