# Supplementary material for: Increased pulmonary blood flow leads to alveolar dysplasia during the early postnatal developmental stage
Source: Cell Biosci. 2025 Nov 24;15:161. doi: 10.1186/s13578-025-01502-x (PMC12642049; doi:10.1186/s13578-025-01502-x)
Supplement: Supplementary file 3 — Supplementary Material 3 [file 13578_2025_1502_MOESM3_ESM.docx]

Supplemental Table S2 Translational potential of neonatal IncPBF model

| Disease / Condition | Relevance to IncPBF Model | Limitations of Existing Models | How the IncPBF Model Addresses the Gap |
| --- | --- | --- | --- |
| Pediatric PAH | Mimics left-to-right shunt physiology (e.g., VSD, PDA) in neonates | MCT/hypoxia models do not recapitulate hemodynamics or neonatal timing | Neonatal ACF induces IPF with preserved early alveolarization stage (P7–P14) |
| Bronchopulmonary dysplasia (BPD) | Alveolar simplification + vascular defects similar to BPD pathology | Hyperoxia models lack cardiovascular triggers | IPF-driven dysplasia resembles multifactorial BPD insult |
| Alveolar capillary dysplasia (ACD) | Features disrupted alveolar–vascular development | Genetic models are limited and non-hemodynamic | Provides an acquired microvascular injury model under flow stress |
