# Supplementary figures and images for: Increased pulmonary blood flow leads to alveolar dysplasia during the early postnatal developmental stage
Source: Cell Biosci. 2025 Nov 24;15:161. doi: 10.1186/s13578-025-01502-x (PMC12642049; doi:10.1186/s13578-025-01502-x)

## Slide 1
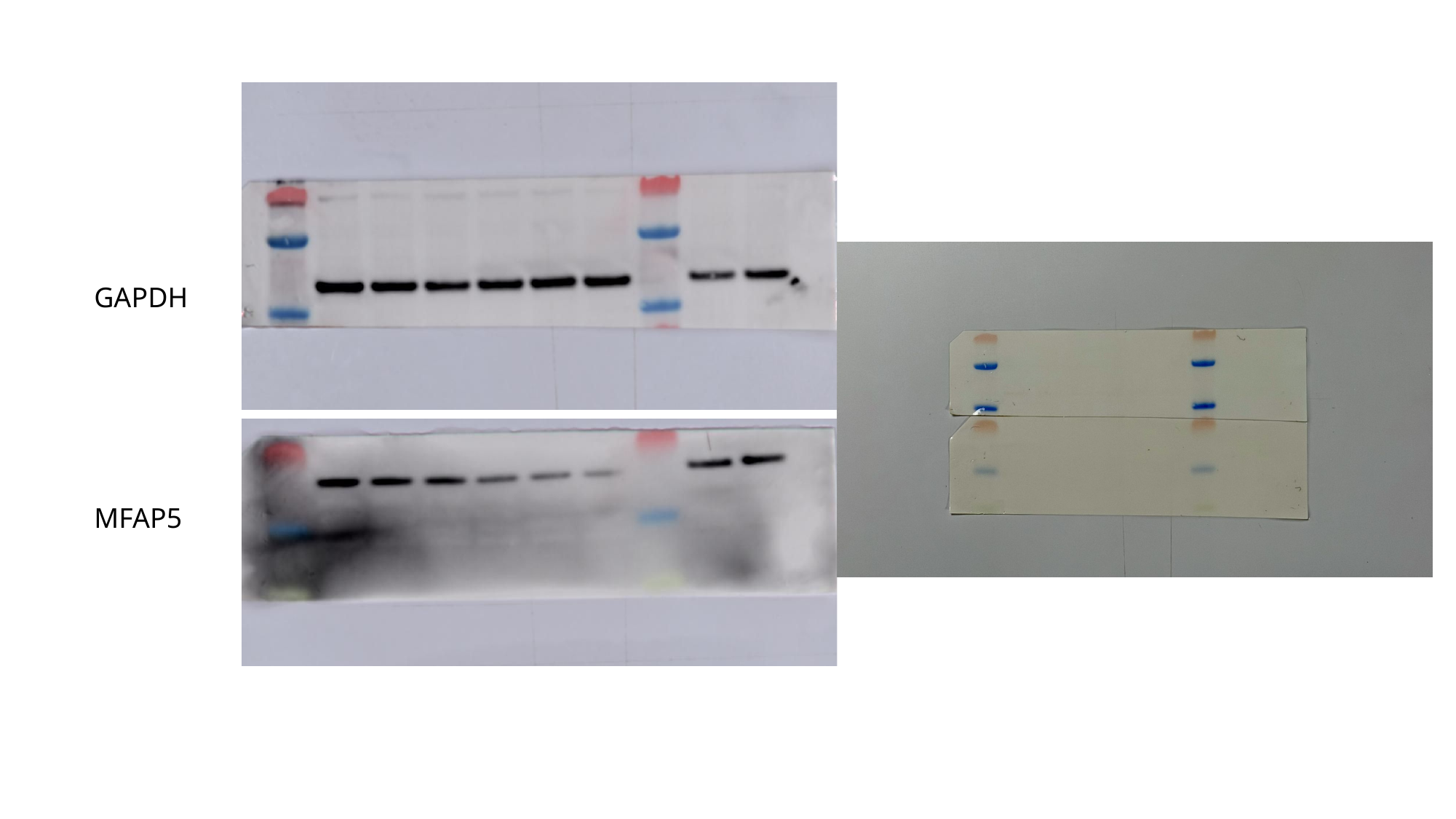

GAPDH
MFAP5

Supplement: Supplementary file 4 — Supplementary Material 4 [file 13578_2025_1502_MOESM4_ESM.pptx]
