# Supplementary material for: Increased pulmonary blood flow leads to alveolar dysplasia during the early postnatal developmental stage
Source: Cell Biosci. 2025 Nov 24;15:161. doi: 10.1186/s13578-025-01502-x (PMC12642049; doi:10.1186/s13578-025-01502-x)

**Supplemental Figures**

**Supplemental Fig. S1 ACF surgery process.** (A) Schematic diagram of puncture site. (B)ACF surgery process: (1-2) Pull small intestine to expose abdominal aorta (AA) and inferior vena cava (IVC). (3) Puncture from the AA into the proximal segment of the IVC at an angle of 30° to 60°. (4) Close the abdomen layer by layer.

**Supplemental Fig. S2 Abdominal ultrasound of the aorta and inferior vena cava.** (A) No pulsatile blood flow in the inferior vena cava (IVC). (B) Pulsatile blood flow in the abdominal aorta (AA).

**Supplemental Fig. S3 IncPBF leads to persistent alveolar dysplasia at P30 and P60.** (A) Representative H&E staining of alveoli in the sham and IncPBF groups. (B) Quantification of MLI.

**Supplemental Fig. S4 Hemodynamic measurements in IncPBF mice.** (A) Doppler flow patterns over time in IncPBF. PA regurgitation was observed at 3M. (B) Two-dimensional measurements of PA parameters. (C) PA regurgitation on color Doppler echocardiography. (D-F) Changes in PA-VTI, PA diameter, and RVSV over time. (G) Histogram of RVSP showed increased RVSP at 2M and 3M after ACF surgery (n=6 per group; Student's t-test; * indicates statistical significance). Abbreviations: P14 = postnatal day 14; P21 = postnatal day 21; PA = pulmonary artery; PVR = pulmonary valve regurgitation; RVSP = right ventricular systolic pressure; M = months; W = weeks. (adopted from Sun S, et al. J Vis Exp. 2023 Jun 9;(196) with the permission of publisher.)

**Supplemental Fig. S5 IncPBF leads to an increase in pulmonary mesenchymal cells**. DAPI (blue); Vimentin (green).

**Supplemental Fig. S6 Heat map of the marker genes of smooth muscle cells/mesenchymal cells and key transcription factors**.

**Supplemental Fig. S7 IncPBF leads to the remodeling of pulmonary small blood vessels.** DAPI (blue); SMA (red).

Supplemental Fig.1


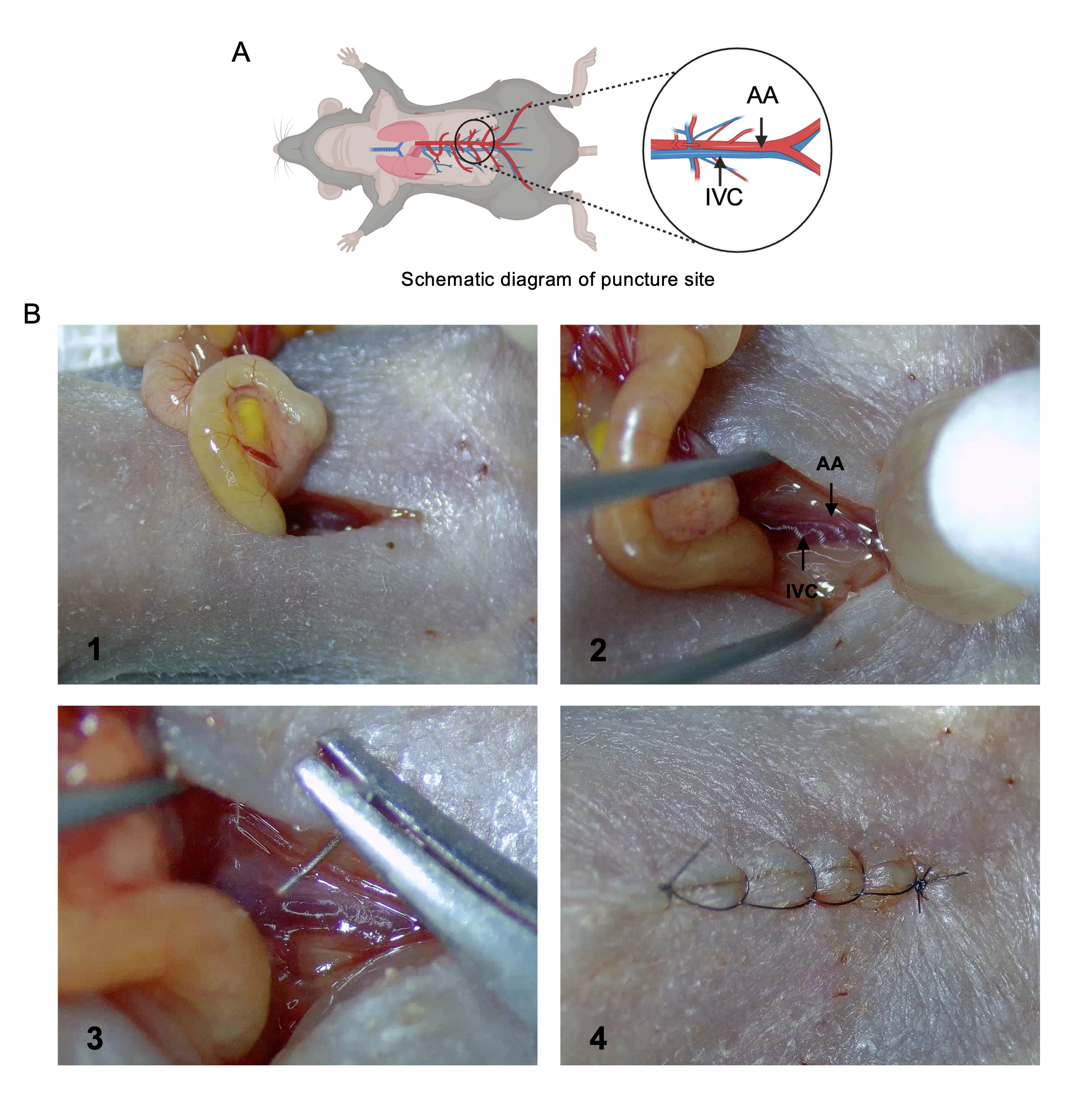


Supplemental Fig.S2


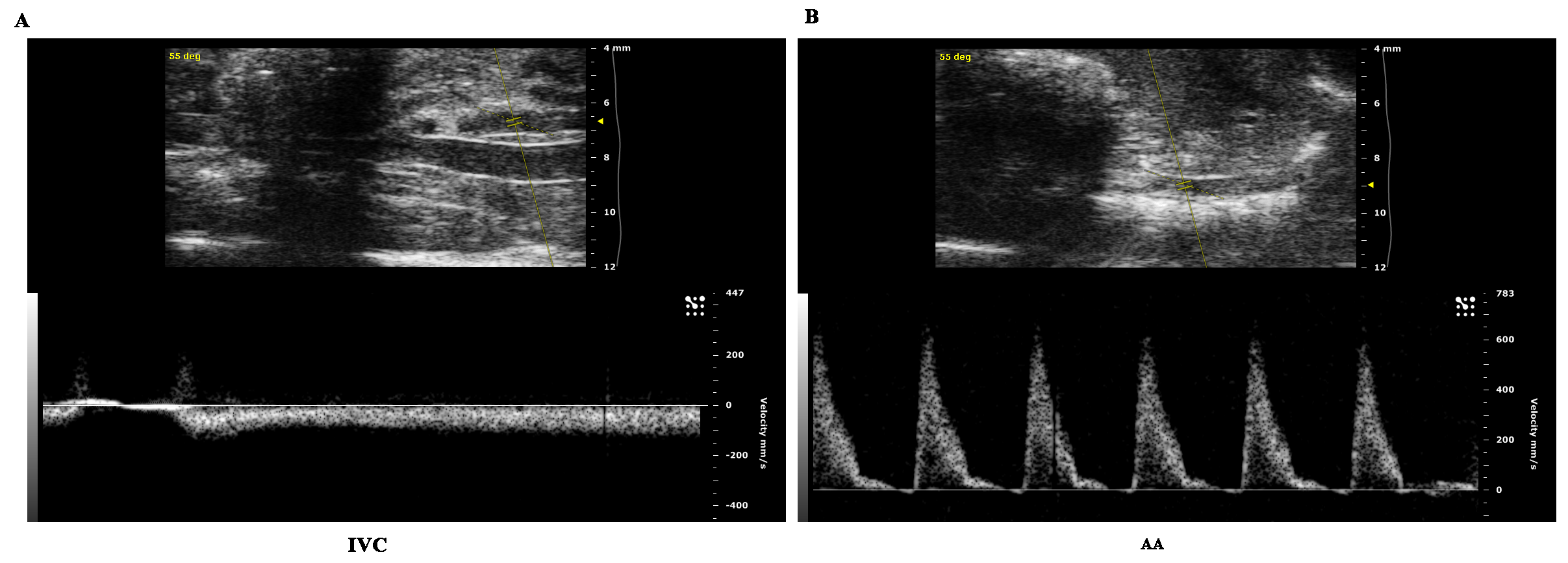


Supplemental Fig.S3


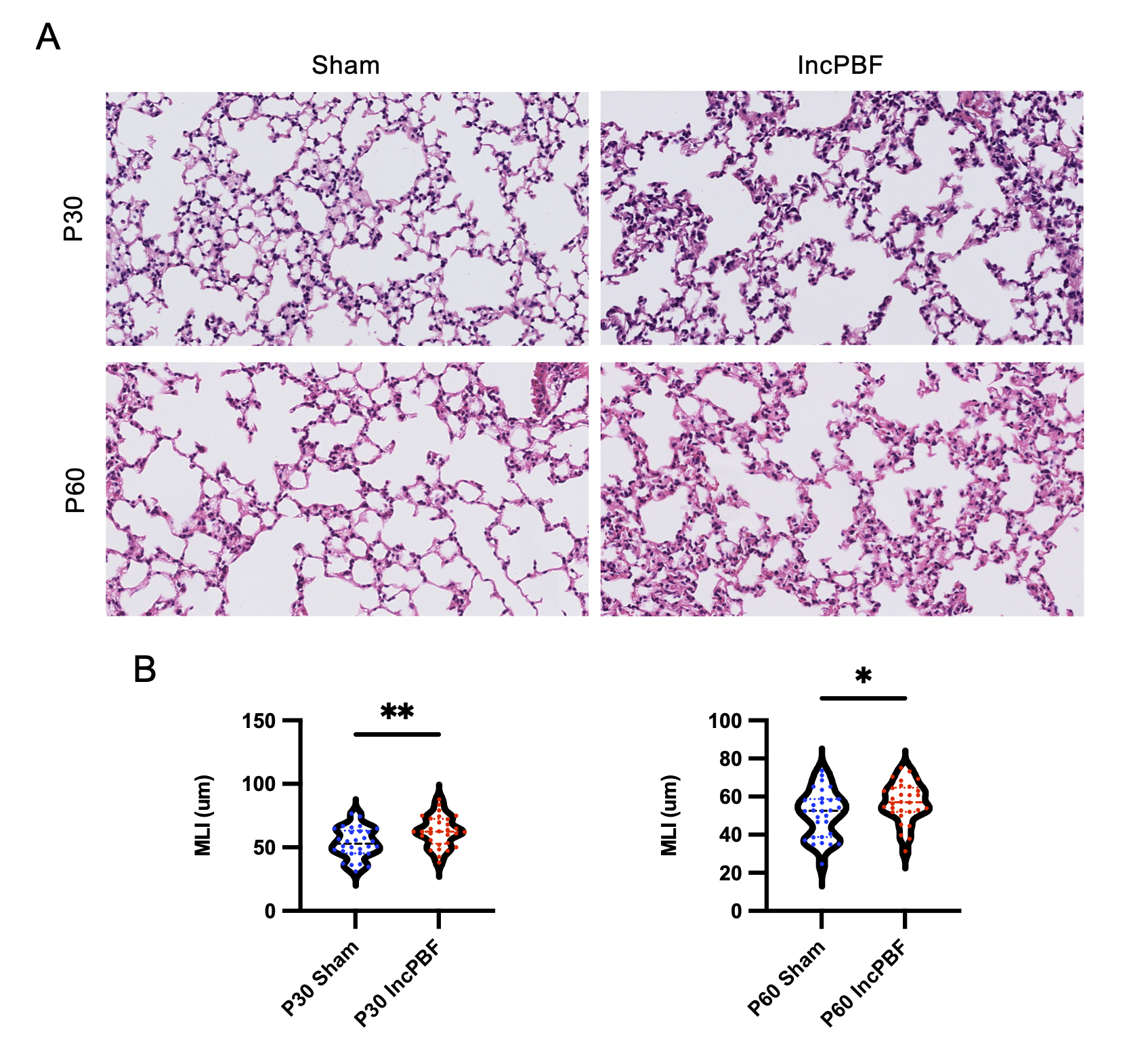


Supplemental Fig.S4


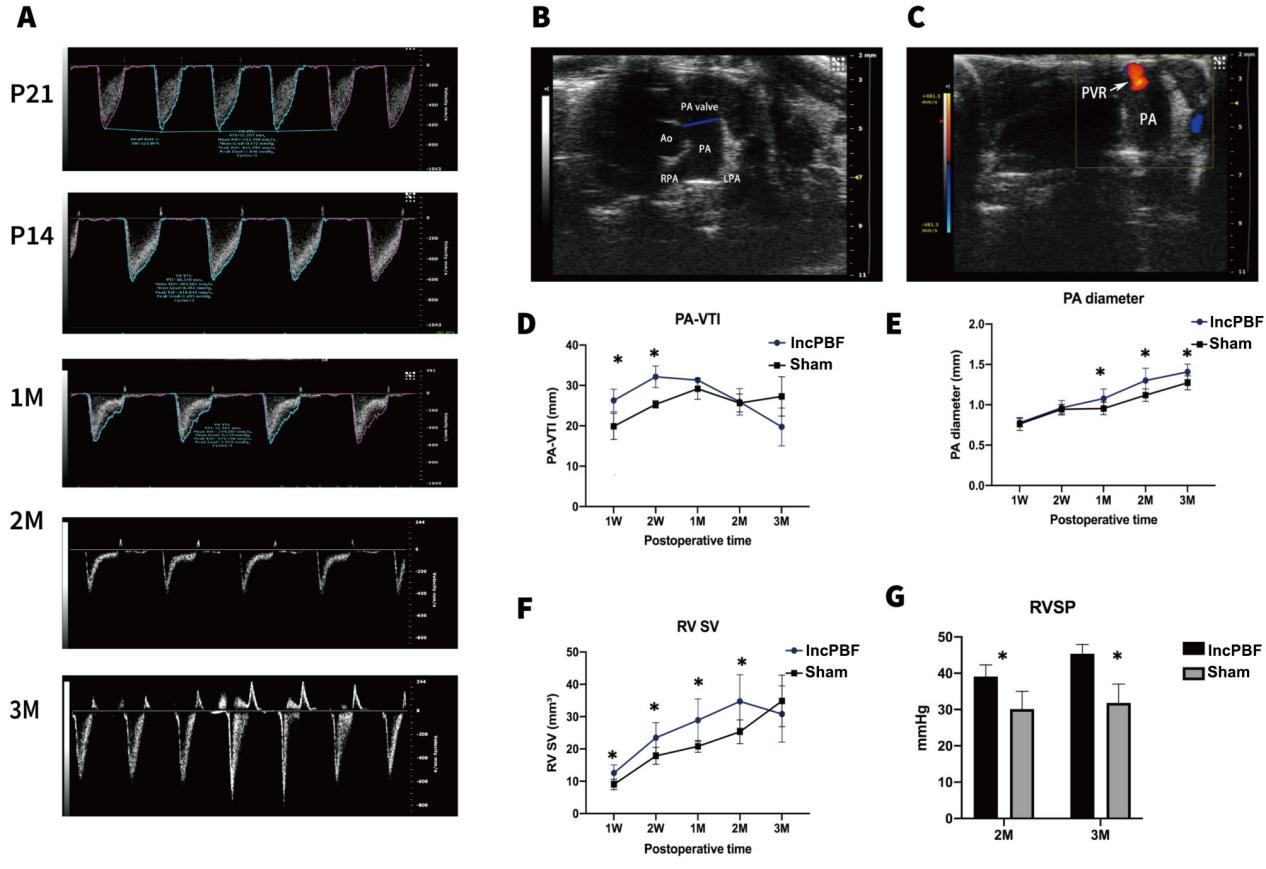


Supplemental Fig. S5


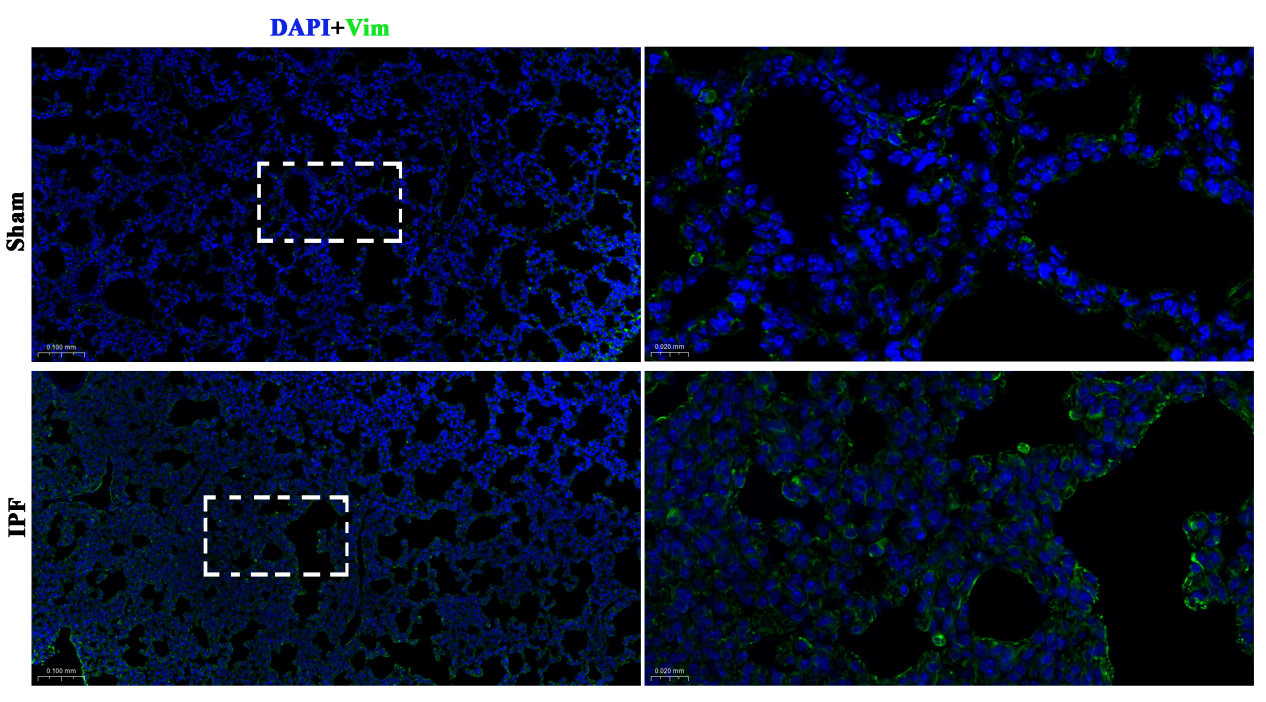


Supplemental Fig.S6


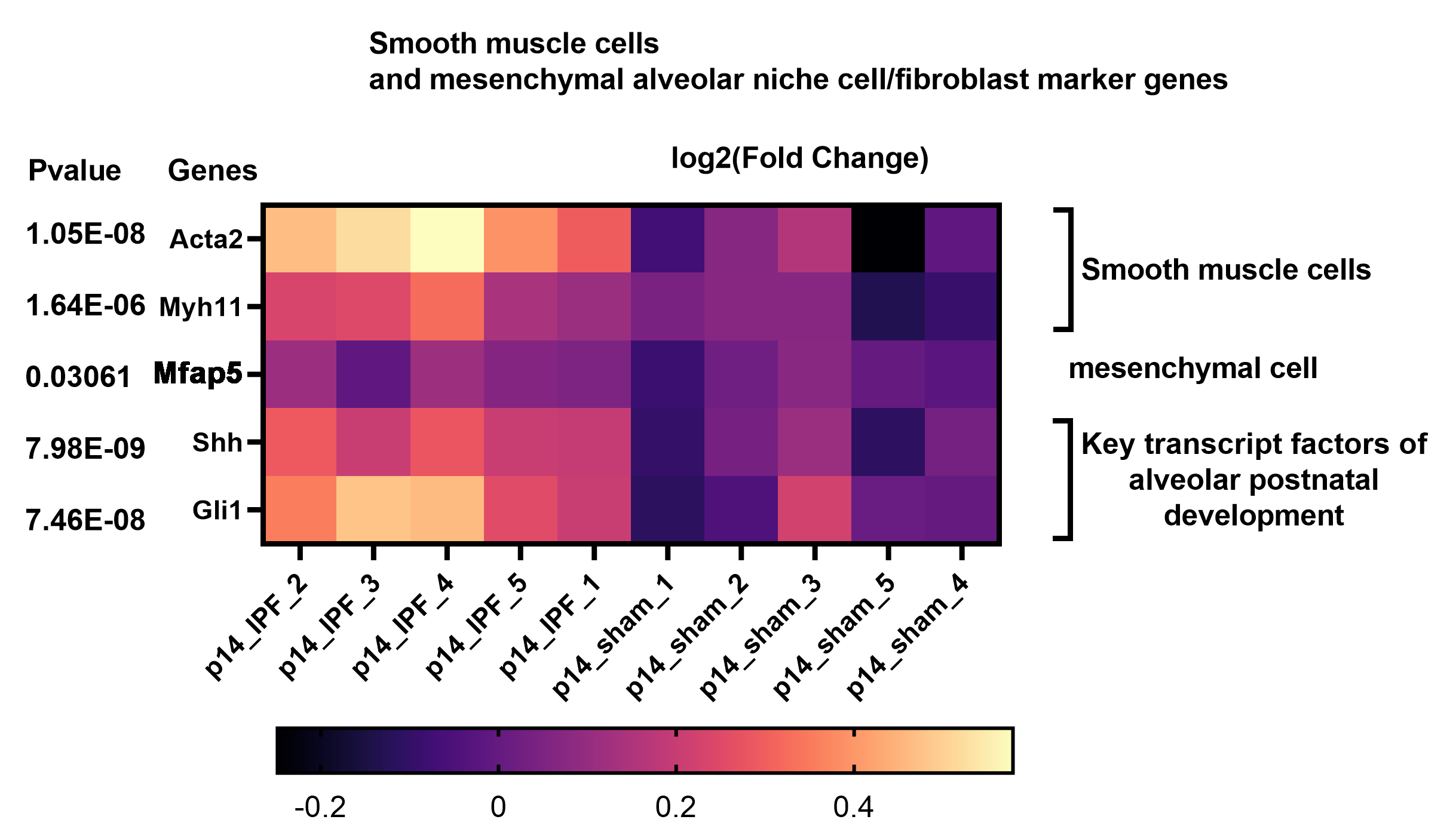


Supplemental Fig.S7


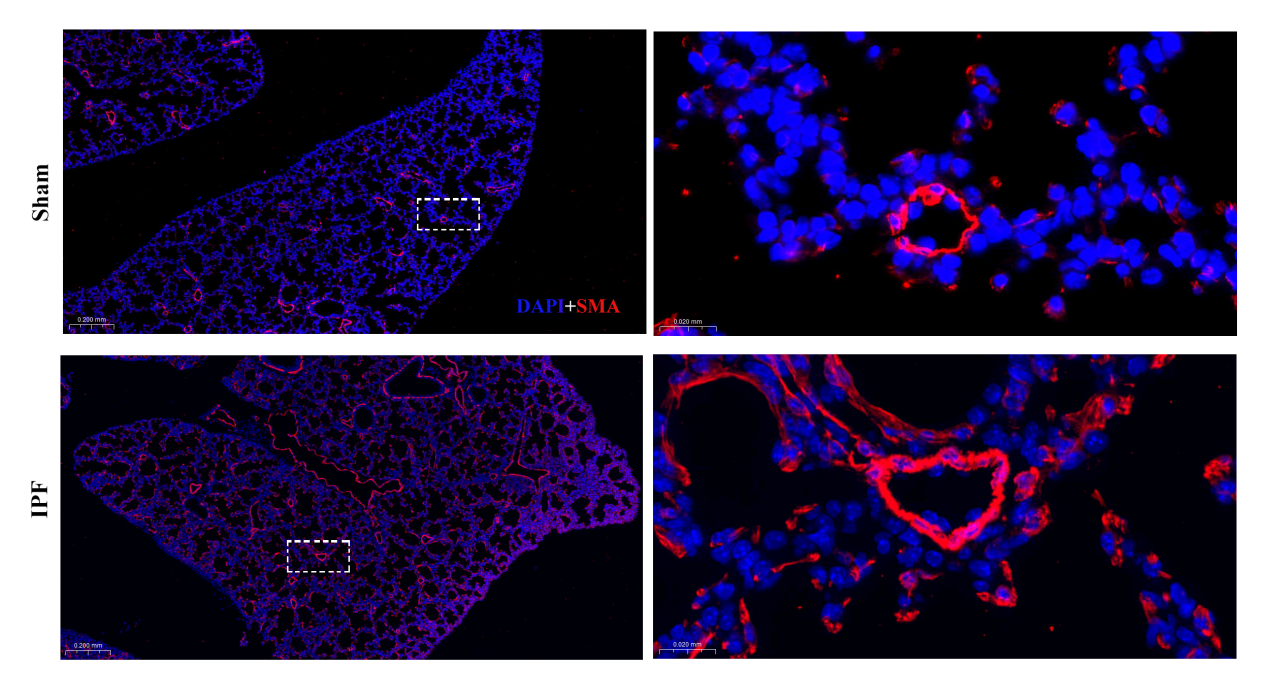

Supplement: Supplementary file 5 — Supplementary Material 5 [file 13578_2025_1502_MOESM5_ESM.docx]
